# Forced Expiratory Volume in One Second Predicts Length of Stay and In-Hospital Mortality in Patients Undergoing Cardiac Surgery: A Retrospective Cohort Study

**DOI:** 10.1371/journal.pone.0064565

**Published:** 2013-05-28

**Authors:** David A. McAllister, Sarah H. Wild, John D. MacLay, Andrew Robson, David E. Newby, William MacNee, J. Alastair Innes, Vipin Zamvar, Nicholas L. Mills

**Affiliations:** 1 Centre for Population Health Sciences, University of Edinburgh, Midlothian, Edinburgh, United Kingdom; 2 MRC Centre for Inflammation Research, University of Edinburgh, Midlothian, Edinburgh, United Kingdom; 3 Respiratory Unit and Respiratory Function Service, Western General Hospital, Midlothian, Edinburgh, United Kingdom; 4 Centre for Cardiovascular Science, University of Edinburgh, Midlothian, Edinburgh, United Kingdom; 5 Department of Cardiothoracic Surgery, Royal Infirmary of Edinburgh, Midlothian, Edinburgh, United Kingdom; Thomas Jefferson University, United States of America

## Abstract

**Objective:**

An aging population and increasing use of percutaneous therapies have resulted in older patients with more co-morbidity being referred for cardiac surgery. Objective measurements of physiological reserve and severity of co-morbid disease are required to improve risk stratification. We hypothesised that FEV_1_ would predict mortality and length of stay following cardiac surgery.

**Methods:**

We assessed clinical outcomes in 2,241 consecutive patients undergoing coronary artery bypass grafting and/or valve surgery from 2001 to 2007 in a regional cardiac centre. Generalized linear models of the association between FEV_1_ and length of hospital stay and mortality were adjusted for age, sex, height, body mass index, socioeconomic status, smoking, cardiovascular risk factors, long-term use of bronchodilators or steroids for lung disease, and type and urgency of surgery. FEV_1_ was compared to an established risk prediction model, the EuroSCORE.

**Results:**

Spirometry was performed in 2,082 patients (93%) whose mean (SD) age was 67 (10) years. Median hospital stay was 3 days longer in patients in the lowest compared to the highest quintile for FEV_1_, 1.35-fold higher (95% CI 1.20–1.52; p<0.001). The adjusted odds ratio for mortality was increased 2.11-fold (95% CI 1.45–3.08; p<0.001) per standard deviation decrement in FEV_1_ (800 ml). FEV_1_ improved discrimination of the EuroSCORE for mortality. Similar associations were found after excluding people with known pulmonary disease and/or airflow limitation on spirometry.

**Conclusions:**

Reduced FEV_1_ strongly predicted increased length of stay and in-hospital mortality following cardiac surgery. FEV_1_ is a widely available measure of physiological health that may improve risk stratification of complex patients undergoing cardiac surgery and should be evaluated for inclusion in new prediction tools.

## Introduction

For more than 40 years cardiac surgery has been performed to relieve symptoms and improve outcomes in patients with coronary artery and valvular heart disease. [1] Whilst improvements in surgical technique have resulted in progressive reductions in operative morbidity and mortality, the aging population and increasing use of percutaneous treatments have resulted in older patients with more co-morbidity being referred for cardiac surgery. [2].

Several prognostic indices have been developed to help patients and clinicians decide whether cardiac surgery is appropriate. The European System for Cardiac Operative Risk Evaluation (EuroSCORE) is a widely used risk prediction tool that comprises seventeen clinical features, [3] and has good discrimination for early and late post-operative mortality. [4] It is recognised that changes in surgical practice over the last decade require these clinical tools to be updated. [5] With increasingly elderly patients being considered for surgical management, markers which reflect general physiological reserve and severity of co-morbid disease may be particularly relevant to prognosis and should be evaluated for inclusion in new risk prediction tools.

Forced Expiratory Volume in one second (FEV_1_) is a robust accurate measure of pulmonary physiology, and is a strong predictor of mortality from cardiovascular, respiratory and other causes, independent of age, gender, race/ethnicity, socioeconomic status, blood pressure, diabetes and cholesterol, and has been found to predict mortality in patients undergoing oesophagectomy.[6]–[8] However, studies directly examining whether FEV_1_ predicts outcomes in cardiac surgery have been limited to small numbers of patients with known pulmonary disease. [9], [10] As such, the relationship between FEV_1_ and clinical outcomes following cardiac surgery has not been established, particularly in patients without known pulmonary disease.

We hypothesised that FEV_1_ would predict length of hospital stay and in-hospital mortality independent of a clinical history of pulmonary disease and other potential confounders, and that the addition of FEV_1_ to the EuroSCORE would improve risk stratification in patients undergoing cardiac surgery.

## Patients and Methods

Database Linkage was approved by Lothian Caldicott Officer and the Chair of Lothian Research Ethics Committee advised that the use of the fully irreversibly anonymised data for this research did not require ethical approval by the full committee.

### Study Population

In a retrospective cohort study we identified all patients aged 40 years or older who underwent coronary artery bypass grafting (CABG) and/or valve repair or replacement surgery from January 1st 2001 to December 31st 2007 at the Royal Infirmary of Edinburgh, Scotland, United Kingdom. Patients who were not resident in the hospital catchment area or who underwent cardiac surgery as an emergency were excluded a priori on the basis that spirometry would not have been performed locally or would not have been feasible prior to surgery. Where patients had more than one operation during the study period, only the first operation was included.

### Clinical Characteristics and Outcomes

Patient characteristics and details of their cardiac surgery, including length of hospital stay and in-hospital mortality, were obtained from medical records and recorded in an electronic cardiac surgical database (TOMCAT Clinical Systems, Philips Healthcare, Amsterdam, The Netherlands) by trained staff at the time of admission. Hospital stay was defined as a single continuous NHS stay following surgery, excluding long-term facilities. In-hospital mortality was defined as any death within this stay. This database is maintained to nationally agreed standards, which includes auditing of randomly sampled medical records and publication of statistics on missing data (www.scts.org/sections/audit/Cardiac/index.html). In an external Scottish Quality Improvement Programme audit in 2008 these data were found to be 98.5% accurate.

An estimate of predicted operative mortality was derived using the EuroSCORE (excluding the following variables which were not included in the linked dataset - active endocarditis, critical pre-operative state, or post-infarct septal rupture). [5](3) The EuroSCORE defines ‘chronic pulmonary disease’ as the long term use of bronchodilators or steroids for lung disease. Patient or clinician reported diagnoses of chronic obstructive pulmonary disease (COPD) and asthma were also recorded. The 2006 Scottish Index of Multiple Deprivation (SIMD) score was obtained from the recorded postal code and patients were stratified based on the national distribution of the SIMD in order to provide an area-based measure of socio-economic status (www.scotland.gov.uk/Publications/2005/10/1893201/32023).

### Spirometry

Height and weight were measured, and spirometry was performed on a wedge bellows spirometer to Association for Respiratory Technology and Physiology/British Thoracic Society standards (Model S, Vitalograph, Buckinghamshire UK) by trained clinical physiologists in one of two pulmonary function laboratories. FEV_1_ percent predicted was calculated using the European Coal and Steel reference equations. [11] Airflow obstruction was defined a priori as an FEV_1_/FVC ratio below 0.7 and FEV_1_ percent predicted less than 80% according to UK national guidance (http://guidance.nice.org.uk/CG12). A broader definition of airflow obstruction for use in sensitivity analyses included all patients with an FEV_1_/FVC ratio below 0.7 regardless of the FEV_1_ percent predicted. We did not measure post bronchodilator spirometry, and therefore in order to estimate the likely prevalence of COPD according to international guidelines (www.goldcopd.com and www.thoracic.org/clinical/copd-guidelines), which require post-bronchodilator spirometry, we used the difference in prevalence of airflow obstruction between pre and post bronchodilator spirometry in the international PLATINO COPD prevalence study [12] to adjust the proportion of patients with FEV_1_/FVC ratio below 0.7 in our cohort.

### Record Linkage

Records from the cardiac surgery database were linked to the spirometry database for the same period. Within a secure health service environment, date of birth, first name and surname were used to link records using Microsoft Access 2000. Personal identifiers and dates were removed following linkage and the irreversibly anonymised database was used for analysis. Where patients had spirometry performed more than once prior to surgery, the measure closest to the date of the operation was selected. Patient selection and matching were completed prior to all analyses.

### Statistical Analysis

For descriptive purposes, characteristics were compared for patients with and without spirometry records and by quintile of FEV_1_ percent predicted, which is widely used in clinical practice. However, use of the current European prediction equations has been criticised on the grounds that they are not contemporary and were not obtained from original data. [13] As such, all formal analyses were performed using FEV_1_ with adjustment for age, sex, height and other covariates as appropriate. Proportional change in length of stay, and the odds ratio for in-hospital mortality were estimated by quintile of FEV_1_, and per one standard deviation decrement in FEV_1_, using linear regression of length of stay (log-transformed) and logistic regression respectively.

Although most epidemiological studies report FEV_1_ as the primary marker of impaired lung function, controversy continues as to whether FEV_1_, FVC or the FEV_1_/FVC ratio is the best predictor of mortality in populations not selected to have chronic lung disease. [14], [15] As such, the analyses were repeated with FVC and the FEV_1_/FVC ratio in addition to and as an alternative to the FEV_1_.

Discrimination of the existing EuroSCORE model was compared to a model including EuroSCORE and FEV_1_ using the area under the curve, [16] and the more sensitive net reclassification and integrated discrimination indices (NRI and IDI respectively). [17] The NRI compares models with and without a new marker of interest to estimate the proportion of patients more appropriately classified minus the proportion less appropriately classified under the new model. The IDI is a global measure of sensitivity weighted by specificity that is more sensitive than the area under the curve. Model calibration was compared using the le Cessie-van Houwelingen normal test statistic for the unweighted sum of squared errors [18].

In sensitivity analyses regression modelling was repeated following stratification by gender, smoking status, type and urgency of operation, and following exclusion of patients with a clinical history of asthma, COPD and long-term use of bronchodilators or steroids for lung disease, or airflow obstruction on spirometry. Generalised additive models were used to explore non-linearity. Where data were missing, multiple imputation was used as the main approach with listwise deletion sensitivity analyses. Analyses were performed in SAS 9.2 (Cary, North Carolina, USA) and R version 2.9.2 (R Foundation for Statistical Computing, Vienna, Austria).

## Results

Records meeting matching criteria were available for 2,082 patients (93%) of the 2,241 patients from the regional cardiac surgery database who met the eligibility criteria. The 159 patients without spirometry were more likely to have required urgent surgery (70 versus 54%, p<0.001), and have a clinical history of COPD (3.8 versus 1.3%, p = 0.02), but were otherwise similar to the 2,082 with matching records.

In patients with spirometry performed the mean (SD) age was 67 (10) years and 1,451 (70%) were male (Table 1). Spirometry was performed in the same year as surgery in 90% of patients, and in the same or previous year in 97% (median (IQR) 3 (1 to 5) months after angiography). Most patients had a history of cigarette smoking with 52% ex-smokers and 15% current smokers, but a clinical diagnosis of COPD was only recorded in 28 (1.3%), asthma in 24 (1.2%) and long-term use of bronchodilators or steroids for lung disease in 20 (1.0%) patients. Airflow obstruction identified on spirometry (FEV_1_/FVC <0.7 and FEV_1_<80% predicted) was present in 318 (15%) patients, of these 80% had an FEV_1_>50% predicted, 17% had an FEV_1_ 30–49% predicted and 3% had an FEV_1_ of <30%.

**Table 1:** Patient characteristics by quintile of FEV_1_ percent predicted.

	Q1	Q2	Q3	Q4	Q5
FEV_1%_ predicted, mean (range)	60 (20–75)	82 (75–88)	93 (88–98)	102 (98–108)	120 (109–177)
n	416	417	416	417	416
Age, years (mean (SD))	69 (9)	66 (10)	66 (10)	66 (10)	68 (10)
Gender, male	68.3	70.0	71.4	72.4	66.4
Deprivation (SIMD)					
First quintile (most deprived)	18	12	9	14	8
Second quintile	24	21	24	22	17
Third quintile	14	17	18	12	15
Fourth quintile	18	23	20	18	22
Fifth quintile (least deprived)	27	28	30	25	39
Height, cm – mean (SD)	168 (9)	168 (9)	168 (9)	168 (9)	166 (10)
Body mass index, kg/m2 (mean (SD))	27.1 (4.9)	28.7 (4.7)	28.1 (4.4)	28.0 (4.6)	27.6 (4.3)
Smoking status					
Never smoked	23	28	34	37	45
Ex-smoker	59	53	50	50	48
Current smoker	19	19	16	13	7
Previous cardiac surgery	8.4	7.9	5.5	2.6	2.4
Diabetes	19	18	17	16	13
Hypertension	59	66	64	63	67
Renal disease	5	1	1	1	1
Previous stroke or TIA	15	9	11	11	6
Extracardiac arteriopathy	11	12	10	8	6
Recent myocardial infarction	63	60	58	60	57
Atrial fibrillation	27	17	13	14	6
Recorded COPD diagnosis	2.6	1.4	1.4	0.5	0.7
Recorded asthma diagnosis	2.2	1.4	1.0	0.2	1.0
Long-term use of bronchodilators or steroids for lung disease	2.4	1.0	0.2	0.7	0.5
Vessels >70% stenosis					
None	31	23	22	23	21
One	8	9	7	6	10
Two	16	17	16	19	18
Three	44	51	54	52	51
Left ventricular ejection fraction					
Normal	61	69	72	72	78
Moderately impaired	28	22	24	25	17
Severely impaired	11	9	5	3	5
Pulmonary hypertension (systolic BP>60 mmHg)	30	27	20	6	11
Type of operation					
CABG	53.6	63.1	67.6	67.9	67.3
CABG and valve	14.2	13.0	12.3	10.3	13.2
Valve	32.2	24.0	20.2	21.8	19.5
Urgent surgery	59.6	52.0	55.3	52.3	50.5

Values are percentage unless stated.

Using the broader definition of airflow obstruction requiring an FEV_1_/FVC ratio below 0.7 regardless of the FEV_1_ percent predicted, 511 (25%) patients had airflow limitation. Adjusting this estimate to account for the fact that a bronchodilator was not administered prior to spirometry [12] an estimated 18% of patients had airflow obstruction according to current international guidelines [19], [20] (Table S1).

Socioeconomic deprivation, urgent surgery, valve procedures, current smoking, diabetes mellitus, stroke, extra-cardiac arteriopathy (a history of claudication, documented carotid artery occlusion or >50% stenosis, or surgery for disease of the abdominal aorta, peripheral vasculature or carotid arteries), left ventricular impairment, pulmonary hypertension (systolic blood pressure >60 mmHg), atrial fibrillation and dyspnoea were more frequent in quintiles with lower FEV_1_ percent predicted than those in higher quintiles, but the proportion of patients with a >70% stenosis in one or more of the main coronary arteries was similar across FEV_1_ percent predicted quintiles (Table 1).

### Length of Stay

Median hospital stay was 3 days longer amongst patients in the lowest quintile for FEV_1_ compared to patients in the highest quintile (1.53-fold longer; 95% CI 1.41–1.67; p<0.001), and this association persisted after adjusting for age, sex, height, body mass index, type of operation, urgency of procedure, deprivation score, smoking status, recent myocardial infarction, extracardiac arteriopathy, diabetes, hypertension, stroke, atrial fibrillation, left ventricular function, asthma, COPD, bronchodilator use, preoperative renal failure, and pre-operative angina and dyspnoea (1.35-fold longer; 95% CI 1.20–1.52; p<0.001) (Table 2).

**Table 2:** Length of stay (LOS) by quintile of FEV_1_.

	Q1	Q2	Q3	Q4	Q5	SD decrement in FEV_1_	P-value
FEV_1_ litres, mean (range)	1.31 (0.35–1.70)	1.95 (1.71–2.20)	2.41 (2.21–2.62)	2.89 (2.63–3.15)	3.57 (3.16–5.80)	(800 ml)	
n	416	417	416	417	416		
LOS in days, median (IQR)	10 (7–15)	8 (7–13)	7 (6–10)	7 (6–9)	7 (6–8)		
Relative LOS unadjusted	1.53	1.40	1.25	1.10	1	1.17	<0.001
(95% CI)	(1.41–1.67)	(1.29–1.52)	(1.15–1.36)	(1.01–1.20)		1.14–1.20	
Relative LOS - model 1	1.53	1.38	1.21	1.07	1	1.17	<0.001
(95% CI)	(1.36–1.71)	(1.25–1.53)	(1.11–1.33)	(0.98–1.16)		1.12–1.21	
Relative LOS - model 2	1.35	1.30	1.16	1.05	1	1.12	<0.001
(95% CI)	(1.20–1.52)	(1.17–1.44)	(1.05–1.27)	(0.96–1.14)		1.07–1.17	

Relative LOS – proportional change in length of stay relative to reference category.

Model 1 - adjusted for age, sex, height and body mass index.

Model 2– as model 2 additionally adjusting for type of operation, urgency of procedure, deprivation score, smoking status, recent myocardial infarction, extracardiac arteriopathy, diabetes, hypertension, stroke, atrial fibrillation, left ventricular function, asthma, COPD, long-term use of bronchodilators or steroids for lung disease, preoperative renal failure, pre-operative angina, pre-operative dyspnoea.

### Mortality

Strong associations were found for in-hospital mortality and FEV_1_ (Table 3). The unadjusted odds ratio (OR) for in-hospital death was 2.58-fold higher (95% CI 1.95–3.41; p<0.001) per standard deviation decrement in FEV_1_ (800 ml). FEV_1_ remained a strong predictor after adjusting for type of operation, smoking status, asthma, COPD, bronchodilator use, pre-operative angina and pre-operative dyspnoea (OR 2.11; 95%CI 1.45–3.08; p<0.001).

**Table 3:** In-hospital mortality by quintile of FEV_1_.

	Q1	Q2	Q3	Q4	Q5	SD decrementin FEV_1_	P-value
FEV_1_ litres, mean (range)	1.31 (0.35–1.70)	1.95 (1.71–2.20)	2.41 (2.21–2.62)	2.89 (2.63–3.15)	3.57 (3.16–5.80)	(800 ml)	
n	416	417	416	417	416		
Mortality, n (%)	36 (8.7%)	16 (3.8%)	12 (2.9%)	3 (0.7%)	4 (1.0%)		
Odds Ratio unadjusted	9.76	4.11	3.06	0.75	1	2.58	<0.001
(95% CI)	(3.44–27.7)	(1.36 12.41)	(0.98–9.57)	(0.17–3.36)		1.95–3.41	
Odds Ratio - model 1	8.61	3.73	2.94	0.74	1	2.53	<0.001
(95% CI)	(2.58–28.81)	(1.13–12.33)	(0.91–9.53)	(0.16–3.36)		1.78–3.60	
Odds Ratio - model 2	5.23	2.76	2.28	0.69	1	2.11	<0.001
(95% CI)	(1.48–18.41)	(0.81–9.36)	(0.69–7.53)	(0.15–3.15)		1.45–3.08	

Model 1 - adjusted for age, sex, height and body mass index.

Model 2 - as model 1 additionally adjusting for type of operation, smoking status, COPD, long-term use of bronchodilators or steroids for lung disease, pre-operative angina, pre-operative dyspnoea.

### Airflow Obstruction on Spirometry and FEV_1_

Adjusting for the same covariates as in the main analyses, FEV_1_ continued to predict hospital stay (800 ml decrement in FEV_1_; 1.26-fold longer; 95% CI 1.08–1.48, p<0.001), and mortality (800 ml decrement in FEV_1_; 2.28 OR; 95% CI; 1.19–4.41; p = 0.01) after excluding individuals with airflow obstruction on spirometry. Similar results were obtained when a broader definition of airflow obstruction was used (FEV_1_/FVC ratio <0.70, regardless of FEV_1_ percent predicted, p<0.001, and p = 0.01 respectively).

**Figure 1:**
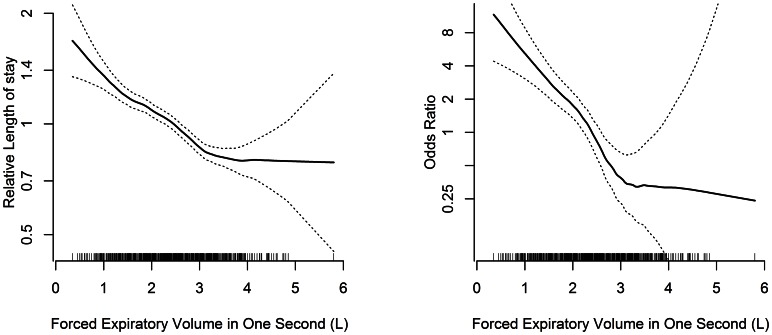
Relationship between FEV_1_, length of stay and mortality. a) Relative length of stay for a given FEV_1_ compared to the length of stay for the mean FEV_1_. b) Odds ratio for in-hospital mortality for a given FEV_1_ compared to the odds ratio for the mean FEV_1_. Estimates were obtained from generalized additive models using a loess smoothing function. A rug plot illustrates the density of the data for given value of FEV_1_. Significance tests for non-linearity were p = 0.13 and p = 0.28 respectively.

**Figure 2:**
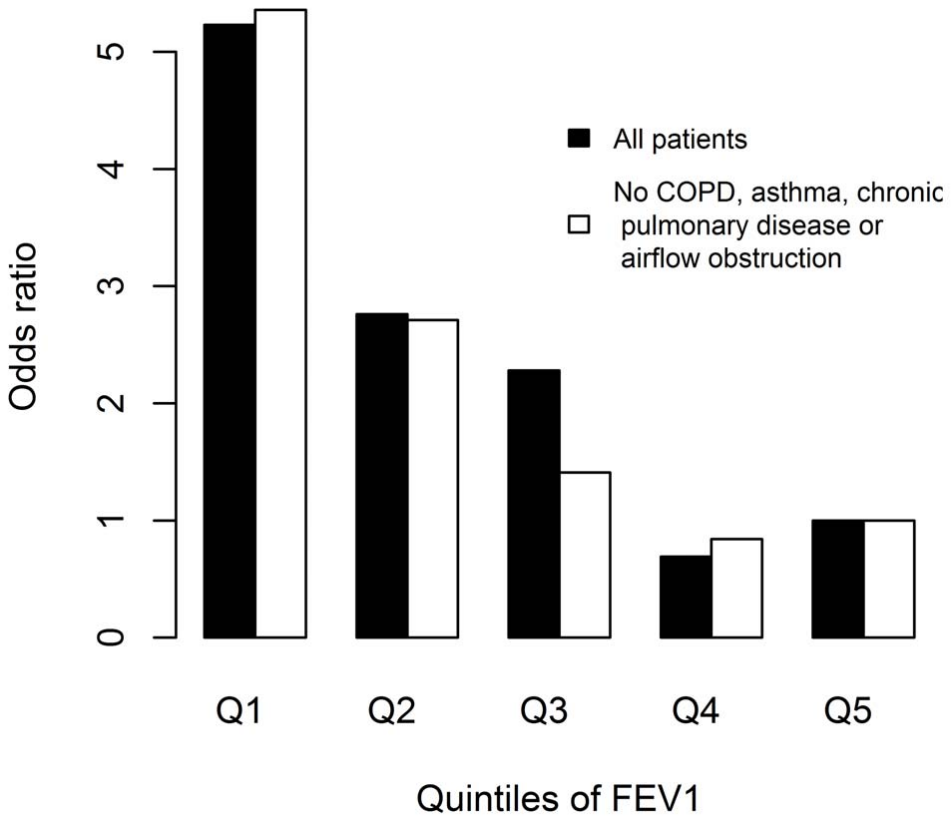
Barplots of association between FEV_1_, and mortality in patients with and without a diagnosis of COPD or asthma, long-term use of bronchodilators or steroids for lung disease, and airflow obstruction on spirometry. Estimates were obtained from generalized linear models adjusting for age, sex, height and body mass index, type of operation, smoking status, pre-operative angina, pre-operative dyspnoea, and, except where these patients were excluded, COPD and long-term use of bronchodilators or steroids for lung disease.

Moreover, there was no suggestion that increased risk was confined to patients with low lung function (Figure 1). There was a suggestion of an inflection for both mortality and length of stay at higher levels of FEV_1_ (4 litres and above) but the data were relatively sparse at this level, the odds ratio for mortality continued to decline, and there was not statistically significant evidence of departure from linearity for either hospital stay or mortality (p = 0.13, and p = 0.28 respectively). Similar results were obtained following exclusion of patients with a clinical history of asthma, COPD, long-term use of bronchodilators or steroids for lung disease, or airflow obstruction (defined as FEV_1_/FVC <0.7) on spirometry (Figure 2).

In addition, similar associations were found among never smokers as among smokers and ex-smokers for length of stay (1.09-fold longer; 95% CI 1.01 to 1.07 and 1.13-fold longer; 95% CI 1.07 to 1.19 respectively) and mortality (OR 2.29; 95% CI 1.00 to 2.58 and OR 2.45; 95% CI 1.55 to 3.95 respectively), indicating that smoking related airflow obstruction is unlikely to be the major driver of the association between pulmonary function and outcomes.

### FVC and the FEV_1_/FVC Ratio

In the fully adjusted models we obtained similar associations for hospital stay (1 SD decrement in FVC; 1.10-fold longer; 95% CI 1.06 to 1.16, p<0.001) and for mortality (1 SD decrement OR 2.04; 95% CI 1.35 to 3.08) for the FVC alone as we did for FEV_1_. Similar, but weaker associations were found for hospital stay and mortality for FEV_1_/FVC alone compared to FEV_1_ (p = 0.006, p = 0.06 respectively).

However, neither the FVC nor the FEV_1_/FVC ratio added significantly to the FEV_1_ for hospital stay (p = 0.97 and p = 0.84 respectively) or mortality (p = 0.12 and p = 0.57 respectively).

### FEV_1_ Compared to the EuroSCORE

FEV_1_ remained strongly associated with mortality after adjusting for the EuroSCORE (OR 1.69; 95% CI 1.23–2.31, likelihood ratio statistic 11, 1 df, p<0.001). The area under the receiver operator characteristic curve (AUC) was higher for EuroSCORE than FEV_1_, but the difference was not statistically significant (AUC 0.78 and 0.74 respectively, p = 0.15). Similarly, the addition of FEV_1_ to the EuroSCORE increased the AUC when compared to the EuroSCORE alone (AUC of 0.80 and 0.78 respectively), but the difference was not statistically significant (p = 0.26).

However, comparing the area under the curve is known to be insensitive, hence some authors recommend the inclusion of more sensitive measures of discrimination (23) such as the Net Reclassification Index (NRI), [17] according to which 27% of patients (95% CI 4–51%, p<0.001) were more appropriately classified with the EuroSCORE alone than with FEV_1_ alone, while 36% of patients (95% CI 12–59%; p = 0.003) were more appropriately classified when FEV_1_ was combined with the EuroSCORE compared to the EuroSCORE alone. The Integrated Discrimination Index yielded similar results for each of these comparisons: EuroSCORE alone versus FEV_1_ alone (IDI 0.02; 95% CI 0.00–0.03; p = 0.01) and EuroSCORE alone versus FEV_1_ in combination with EuroSCORE (IDI = 0.01; 95% CI 0.00–0.02; p = 0.006). Model calibration was also better with FEV_1_ and EuroSCORE combined (z = 0.14; p = 0.89) than with the EuroSCORE alone (z = 1.27, p = 0.21).

### Sensitivity Analyses

After stratifying by operation type similar associations were found for length of stay (800 ml decrement in FEV_1_; 1.10-fold longer [95% CI 1.01 to 1.21] and 1.12-fold longer [95% CI 1.07 to 1.17] for patients with and without valve surgery respectively) and mortality (1 SD decrement; OR 2.58 [95% CI 1.59 to 4.29] and OR 1.88 [95% CI 0.95 to 3.69] for patients with and without valve surgery respectively). Nor did stratification for urgency of procedure or gender reveal evidence of effect measure heterogeneity. Repeating analyses without adjustment for height yielded very similar associations for FEV_1_ for hospital; stay and mortality. Results obtained using listwise deletion were similar to those obtained using multiple imputation.

## Discussion

FEV_1_ is a strong predictor of longer hospital stay and in-hospital mortality following elective and urgent cardiac surgery. These associations persist after adjusting for multiple covariates including a clinical diagnosis of COPD, and airflow obstruction on spirometry. Addition of FEV_1_ to the current and most widely used peri-operative risk prediction model, the EuroSCORE, improved discrimination and calibration for in-hospital mortality.

COPD is the commonest cause of reduced FEV_1_, [21] and is an established risk factor for in-hospital and long-term mortality following cardiac surgery.[22]–[24] Two recent studies reported that FEV_1_ was associated with poorer outcome in patients with chronic lung disease undergoing cardiac surgery, but due to the small number of patients with measured FEV_1_ (n<150), linearity was not assessed and adjustment for potential confounders was limited. [9], [10] Abadag et al [25] found that pulmonary function predicted increased mortality and length of stay in patients with cardiac surgery, but less than 45% of patients had spirometry measured, with the decision to measure pulmonary function having been made at each clinician’s discretion, making selection bias likely. Moreover, no comparisons within individuals with normal lung function were made. We examined the association between FEV_1_ and both hospital stay and in-hospital mortality across the spectrum of lung function, and found no evidence for a plateau at least until FEV_1_ was moderately high (3–4 litres), and that FEV_1_ predicted hospital stay and mortality even after excluding patients with any evidence of airflow obstruction on spirometry. This association persisted after controlling for other potentially important confounders including age, sex, cardiovascular risk factors and socioeconomic status.

In order to explore the potential usefulness of FEV_1_ for inclusion in risk prediction tools, we also examined the effect of the addition of FEV_1_ on discrimination (distinguishing patients with and without adverse events) and calibration (precision of estimates of risk of adverse events). [26] In a recent review of nineteen published risk prediction models the EuroSCORE was found to be the best discriminator for 30-day and one year mortality after cardiac surgery. [4] We found that FEV_1_ when combined with EuroSCORE improved discrimination compared to EuroSCORE alone. The area under the curve was higher, although the difference was not statistically significant, while the more powerful Net Reclassification Index was highly statistically significant, and estimated that over a third of patients would be assigned a more appropriate risk of in hospital death when FEV_1_ was combined with the EuroSCORE compared to the EuroSCORE alone. Calibration was also improved on adding FEV_1_ to the EuroSCORE. FEV_1_ appears to have additional value, beyond identifying patients with chronic lung disease, as an independent predictor of outcome in patients undergoing cardiac surgery.Moreover, FEV_1_ is not a costly new biomarker, but rather a widely available physiological measurement which we found was routinely performed in over 90% of patients undergoing elective and urgent cardiac surgery. FEV_1_ remains stable over time and can therefore be measured at an early stage of evaluation prior to cardiac surgery [27] and is therefore an attractive candidate for inclusion in prognostic models.

COPD particularly when mild or moderately severe is commonly under-diagnosed in primary care, [18] and the same appears to be true for patients undergoing cardiac surgery. Fewer than 2% had known COPD, lower than the 5% we would have expected based upon the age-sex distribution of our patients and national statistics on the proportion of patients diagnosed with COPD in this region of Scotland (www.isdscotland.org/isd/3707.html). Patients with clinically evident COPD may be less frequently selected for cardiac surgery. Nevertheless, fifteen percent had airflow obstruction on spirometry and a significant proportion of these are likely to have had COPD. As such, under-treatment of mild to moderate COPD (with, for example inadequate inhaled therapy) may contribute to the excess mortality associated with low FEV_1_, and the routine recording of spirometry during pre-assessment for surgery may improve treatment and clinical outcomes in these patients.

Undiagnosed COPD is unlikely to be the primary cause of excess morbidity and mortality since FEV_1_ predicted outcomes similarly well in patients without airflow obstruction, and among never smokers. Furthermore, similar associations were found for FVC as for FEV_1_, and the FEV_1_/FVC ratio did not have additional power over FEV_1_ alone. Reductions in FEV_1_ (and FVC) may reflect both increased exposure and susceptibility to pro-inflammatory factors, such as cigarette smoking, environmental pollution and microbial infection. FEV_1_ may also be influenced by pulmonary congestion secondary to valvular heart disease related which can cause both obstructive and restrictive abnormalities in pulmonary function. [28].

Alternatively, as previously suggested, reduced FEV_1_ may simply be an indicator of the patients’ capacity to withstand the insult of major surgery, reflecting their respiratory reserve, nutritional status or muscle strength. [14], [15] In the general population FEV1 predicts a range of adverse outcomes including, but not limited to cardiovascular, respiratory and even psychiatric illness. [6] Moreover, pulmonary function as measured by spirometry depends not only on lung characteristics such as airway diameter and lung elasticity, but also on skeletal muscle strength [29] (in the diaphragm and intercostal muscles) while pulmonary function also improves following bariatric surgery. [30] Therefore, we believe that FEV1 (and FVC) is likely to reflect the patient’s general capacity to withstand the insult of major surgery, and not just respiratory reserve.

FEV1 and FVC were highly correlated, and performed similarly in predicting length of hospital stay and mortality. However, the FEV1/FVC ratio was less strongly associated with these outcomes. As such, while FEV1/FVC is required, by definition to diagnose COPD, it appears be less useful than FEV1 and FVC as a prognostic marker following cardiac surgery, particularly if a single cut-off value (such as FEV1/FVC <0.7) is used. Therefore, cardiac surgery departments should consider recording the FEV1 and FVC in addition to the FEV1/FVC ratio. Although further multi-centre studies will be required to confirm that future cardiac surgery scores should include pulmonary function measures. Future studies examining cause-specific mortality and complication rates will also allow us to explore the mechanisms underlying the associations with FEV_1_ and mortality and length of stay.

### Limitations

Not all patients had FEV_1_ measured immediately prior to surgery. However, most patients had FEV_1_ measured as part of their surgical pre-assessment, and the decline of FEV_1_ in both the general population and patients with COPD is low (approximately 30 ml/year [27], [31]) Changes in FEV_1_ between pre-assessment spirometry and the date of surgery, for example because patients are started on bronchodilator therapy, are likely to underestimate the strength of association with mortality and length of stay.

We did not record all known prognostic markers at the time of the matching and anonymisation, and did not include active endocarditis, critical pre-operative state, or post-infarct septal rupture in our EuroSCORE calculation. However, these clinical variables are present in less that 0.1% of patients undergoing urgent cardiac surgery in our regional centre (results not shown) and similar associations for FEV_1_ and length of stay and mortality were found even when restricting the analysis to elective surgery, making confounding by these ‘critical illness’ variables unlikely.

The purpose of this study was not to estimate the prevalence of under-diagnosis of COPD. However, we report the prevalence of airflow obstruction using UK COPD guidance current to the year of surgery for our patients (FEV_1_/FVC <0.7 and FEV_1_ less than 80% predicted). [32] Updated UK guidance and international guidelines [19], [20] do not require an FEV_1_ less than 80% predicted, but do stipulate the use of post bronchodilator spirometry. Nevertheless, we were able to adjust for the use of non-post bronchodilator spirometry using data from the international PLATINO study [12] to estimate that approximately 18% of patients are likely to have had post-bronchodilator obstruction, and therefore meet the current international criteria for diagnosis of COPD. However, importantly FEV_1_ predicted mortality and morbidity as well in patients without airflow obstruction (FEV_1_/FVC<0.7 and any report of asthma or COPD), and in never smokers as in the full cohort (Figure 2), suggesting that FEV_1_ is more likely to be a marker of frailty than solely a marker of airflow obstruction.

### Conclusions

FEV_1_ is a strong and independent predictor of length of stay and in-hospital mortality following elective and urgent cardiac surgery, and improves the discrimination of existing risk prediction tools. Spirometry is a widely available non-invasive measure of physiological reserve that is commonly performed in older patients undergoing major surgery. FEV_1_ should be formally evaluated for inclusion in new cardiac surgery risk prediction tools with the goal of improving clinical outcomes in complex patients undergoing cardiac surgery.

## Supporting Information

Table S1 **Comparison of prevalence of airflow obstruction (FEV1/FVC <0.7, ie COPD) in cardiac surgery patients compared to the prevalence of airflow obstruction with and without administration of a bronchodilator in an international prevalence study.** BD – bronchodilator PLATINO – burden of obstructive lung disease. PLATINO defined low-risk subjects (a total of 1895) as patients who were asymptomatic, with low cigarette smoke exposure (<10 pack years, <200 hour-years of biomass smoke, <5 years exposed to dust) and who had no reported asthma or COPD. The remaining subjects were considered high-risk. We defined low risk as never smokers, and high risk as all other patients. * For each stratum the prevalence of airflow obstruction among the cardiac surgery patients was multiplied by the ratio of pre to post bronchodilator defined airflow obstruction from the PLATINO study to obtain the estimated prevalence of airflow obstruction on post-bronchodilator spirometry.
(DOCX)
