# Supplementary material for: Forced Expiratory Volume in One Second Predicts Length of Stay and In-Hospital Mortality in Patients Undergoing Cardiac Surgery: A Retrospective Cohort Study
Source: PLoS One. 2013 May 28;8(5):e64565. doi: 10.1371/journal.pone.0064565 (PMC3665784; doi:10.1371/journal.pone.0064565)
Supplement: Table S1 — Comparison of prevalence of airflow obstruction (FEV1/FVC <0.7, ie COPD) in cardiac surgery patients compared to the prevalence of airflow obstruction with and without administration of a bronchodilator in an international prevalence study. BD – bronchodilator PLATINO – burden of obstructive lung disease. PLATINO defined low-risk subjects (a total of 1895) as patients who were asymptomatic, with low cigarette smoke exposure (<10 pack years, <200 hour-years of biomass smoke, <5 years exposed to dust) and who had no reported asthma or COPD. The remaining subjects were considered high-risk. We defined low risk as never smokers, and high risk as all other patients. * For each stratum the prevalence of airflow obstruction among the cardiac surgery patients was multiplied by the ratio of pre to post bronchodilator defined airflow obstruction from the PLATINO study to obtain the estimated prevalence of airflow obstruction on post-bronchodilator spirometry. (DOCX) [file pone.0064565.s001.docx]

|  |  | PLATINO study | | Cardiac surgery patients | |
| --- | --- | --- | --- | --- | --- |
| Age band | High risk/low risk | Pre-BD | Post-BD | Spirometry measured without BD | Estimated post-BD* |
| 40–45 | High | 12.7 | 6.5 | 15.6 | 8.0 |
| 40–45 | Low | 5.4 | 2.9 | 11.1 | 6.0 |
| 45–50 | High | 17.2 | 10.4 | 7.7 | 4.6 |
| 45–50 | Low | 7.7 | 4.4 | 4.5 | 2.6 |
| 50–60 | High | 28.1 | 18.7 | 19.9 | 13.3 |
| 50–60 | Low | 14.5 | 7.6 | 6.8 | 3.5 |
| 60–70 | High | 43.4 | 30.3 | 27.6 | 19.4 |
| 60–70 | Low | 29.8 | 17.9 | 14.4 | 8.7 |
| >70 | High | 50.3 | 38.7 | 37.1 | 28.6 |
| >70 | Low | 38 | 27.1 | 20.5 | 14.5 |
